# Laparoscopic surgery in a child with a failing Fontan circulation

**DOI:** 10.1002/anr3.12217

**Published:** 2023-03-03

**Authors:** A. Anagnostopoulos, Y. Boegli, M. Perez, A. de Buys Roessingh, S. Di Bernardo, S. Mauron

**Affiliations:** ^1^ Department of Anaesthesiology Lausanne University Hospital Lausanne Switzerland; ^2^ Paediatric Anaesthesia Unit Lausanne University and Lausanne University Hospital Lausanne Switzerland; ^3^ Paediatric Intensive Care Unit Lausanne University and Lausanne University Hospital Lausanne Switzerland; ^4^ Department of Infant and Adolescent Surgery Lausanne University and Lausanne University Hospital Lausanne Switzerland; ^5^ Paediatric Cardiology Unit Lausanne University and Lausanne University Hospital Lausanne Switzerland

**Keywords:** failing Fontan, laparoscopic surgery, pulmonary hypertension

## Abstract

A child with early failure of a Fontan circulation was listed for cardiac transplantation and then developed a subhepatic abscess. Surgical drainage was deemed necessary after the failure of an attempted percutaneous procedure. Following a multidisciplinary discussion, a laparoscopic technique was chosen to optimise postoperative recovery. To our knowledge, the literature does not describe any case of laparoscopic surgery in a patient with a failing Fontan circulation. This case report highlights the physiological variations involved with this management strategy, discusses the implications and risks, and offers some recommendations.

## Introduction

Francis Fontan achieved the first atriopulmonary connection in 1968 in a child with tricuspid atresia [1], and the technique has evolved since then. The peri‐operative mortality linked to the Fontan procedure is currently as low as 1% in selected series [2], but failing Fontan circulation still occurs, with a mortality rate close to 50% [3]. The causes are linked either to ventricular failure, an altered pulmonary vascular bed or insufficient drainage [4, 5]. The need for non‐cardiac surgery in these patients is a challenge for anaesthetists and raises the question of the feasibility of using a laparoscopic technique. This approach has already been widely discussed in stable Fontan physiology [6], however not in the failing Fontan. In this report, we discuss the multidisciplinary management of a patient with a failing Fontan circulation with a subhepatic abscess requiring surgery.

## Case description

A 2‐year‐old child, born with hypoplastic left heart syndrome, underwent an extracardiac surgical fenestrated connection for completion of Fontan circulation. One week after surgery, he developed a chylothorax and a cast bronchitis with hemodynamic instability and respiratory failure. Severe hypoxemia indicated a need for extracorporeal membrane oxygenation (ECMO). A new, larger (8 mm) fenestration between the innominate vein and the right atrium was performed, and as a result the pulmonary and systemic venous pressures decreased. A tracheostomy along with pulmonary vasodilation with sildenafil and bosentan administration allowed progressive ventilatory weaning. In the meantime, the child was listed for a cardiac transplant. He then developed fever and an inflammatory syndrome. Computerised tomography imaging revealed a collection between the left hepatic lobe and the anterior gastric wall (9 cm × 5 cm × 4 cm). Surgical drainage was deemed necessary as percutaneous drainage was not sufficient to evacuate the purulent collection. A pre‐operative transthoracic echocardiogram showed trivial tricuspid insufficiency, normal systolic single ventricle function, no obstruction in the cavopulmonary connections and unobstructed flow in the innominate vein – right atrium conduit.

**Figure 1:**
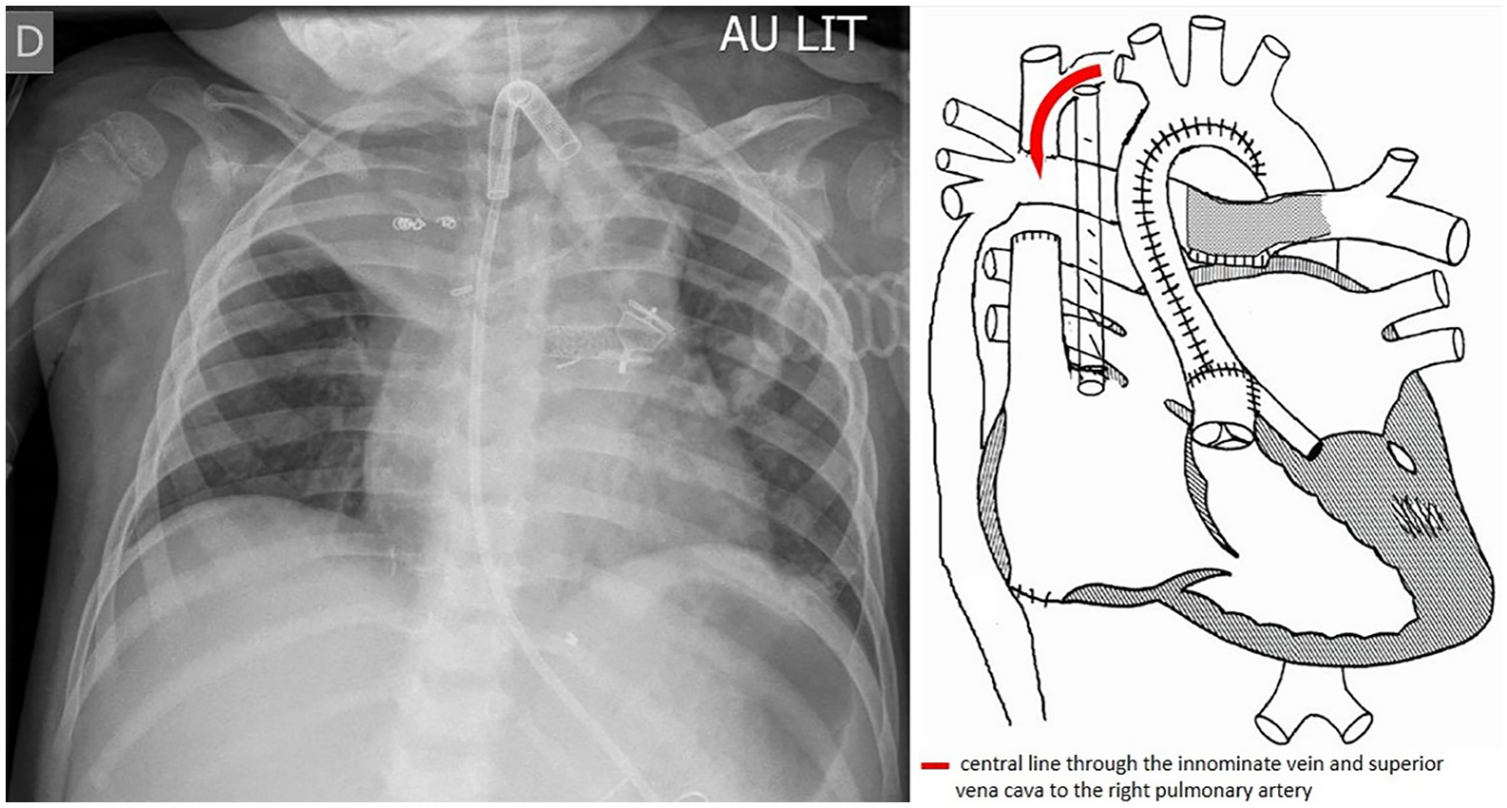
The location of the peripherally inserted central catheter used to monitor pulmonary pressures. The red line demonstrates the position of the central line through the innominate vein and superior vena cava to the right pulmonary artery.

In the operating theatre the child received 2 l.min^−1^ oxygen via his tracheostomy cannula, with a positive end‐expiratory pressure (PEEP) of 5 cmH_2_O. The S_p_O_2_ was 72%, the arterial blood pressure 120/88 mmHg and the heart rate 108 beats.min^−1^. Cerebral oxygenation was monitored with near infrared saturation (NIRS). Intravenous induction of general anaesthesia was with midazolam 0.075 mg.kg^−1^, ketamine 0.75 mg.kg^−1^ and fentanyl 2 μg.kg^−1^. Spontaneous ventilation was maintained, under sevoflurane anaesthesia (end‐tidal 1.5–2%), with intermittent support of the mechanical ventilator. A radial artery line was placed as well as a trans oesophageal echocardiography (TOE) probe. Pulmonary arterial pressures were monitored via a peripherally inserted central catheter (PICC) that was already in place. The PICC travelled through the innominate vein to the superior vena cava, and then directly into the right pulmonary artery due to the Fontan anastomosis, allowing for pulmonary pressure monitoring (Fig. 1). Before initiation of laparoscopy, atracurium 0.5 mg.kg^−1^ was administered intravenously for muscle relaxation and the ventilation was set to pressure control (19 cmH_2_O and no PEEP). The pulmonary arterial pressures increased from 19 mmHg with spontaneous ventilation to 31 mmHg on pressure control. The systolic arterial blood pressure dropped to 80 mmHg; intravenous Ringers Lactate (10 ml.kg^−1^) was administered, and a noradrenaline infusion (0.1 μg.kg.min^−1^) was commenced, with return to baseline blood pressure.

Pneumoperitoneum was established with CO_2_, to a maximum pressure of 9 mmHg. The maximum end‐tidal CO_2_ recorded was 5.87 kPa, correlating to 7.86 kPa on arterial blood gas sampling. Invasive pulmonary arterial pressures progressively rose to a maximum of 39 mmHg. The S_p_O_2_ throughout the procedure oscillated between 72 and 79% with a F_I_O_2_ of 0.8, correlated to arterial oxygen saturation between 68 and 76%. Cerebral NIRS was stable between 62 and 69%. After successful completion of the surgery (total surgical time of 80 min), the child was transferred to the paediatric intensive care unit (PICU) and kept on patient‐triggered inspiratory pressure support ventilation (PEEP 5 cmH_2_O, inspiratory support 6 cmH_2_O and 0.8 F_I_O_2_) (Table 1).

**Table 1:** Vital signs at key milestones during surgery.

	Induction of anaesthesia	Initiation of IPPV	Pneumoperitoneum	Arrival at PICU
S_p_O_2_ (%)	78	74	75	54
ET_CO2_ (kPa)	‐	6.4	5.73	5.87
Systolic arterial pressure (mmHg)	110	92	96	88
Diastolic arterial pressure (mmHg)	66	64	48	40
Pulmonary arterial mean pressure (mmHg)	19	31	37	31
Cerebral oxygen saturation (%)	‐	69	69	68
pH	7.4	7.25	7.22	7.26
PaO_2_ (kPa)	4.5	6.67	5.86	4.9
PaCO_2_ (kPa)	5.6	6.8	8	7.86
Lactate (mmol.l^−1^)	0.75	0.86	1.05	1.41
Insufflation abdominal pressure (cmH_2_O)	‐	‐	10	‐

IPPV: intermittent positive pressure ventilation; ET_CO2_: end tidal CO_2_; PICU: paediatric intensive care unit.

Shortly after arrival in the PICU, the child's oxygen saturation gradually dropped to 54% requiring an increase in ventilatory support (PEEP 8 cmH_2_O, inspiratory support 10 cmH_2_O, F_I_O_2_ 1.0) and finally the introduction of inhaled nitric oxide (NO_i_) at 10 ppm, which increased the S_p_O_2_ back to baseline of approximately 75%. Over the next 12 h, the FiO_2_ was gradually lowered to 0.3 and NO_i_ titrated down. Restoration of the former clinical condition was achieved after a few days, with diuretic and pulmonary vasodilator treatment.

## Discussion

The Fontan circulation physiology has been widely discussed in the literature. Positive intrathoracic pressure during intermittent positive‐pressure ventilation (IPPV) can lead to increased resistance to the blood flow to the lungs, with dramatic consequences. A laparoscopic surgical technique can exacerbate this physiological disturbance, posing an even greater obstruction to blood reaching the lungs. Abdominal insufflation with CO_2_ is known to increase pulmonary vascular resistance (PVR) through the rise in intrathoracic pressures, atelectasis and hypercapnia [7], and can increase systemic vascular resistance (SVR) and reduce cardiac output.

In a regular Fontan circulation, PVR has to be carefully monitored, and kept as low as possible, as all of the cardiac output eventually flows through the lungs. In a failing Fontan circulation, the passive venous blood return to the lungs is highly dependent on both SVR and PVR. Any increase in SVR diminishes the cardiac output, whereas an increase in PVR impairs pulmonary blood flow, leading to redirection of the blood flow through the fenestration to the right atrium. This might cause a dramatic desaturation and increase the risk of ischemic lesions. It is therefore critical to monitor S_p_O_2_, and any desaturation should be interpreted as arising secondary to an increase in PVR, with more blood shunting the lungs through the fenestration. At the other end of the system, a normal diastolic and systolic function of the single ventricle in a Fontan circulation plays a very important role in decreasing the tele‐diastolic pressure and creating a pressure gradient driving blood through the pulmonary vasculature. The use of TOE in our case was required to monitor the systolic function during the procedure. It could also have been useful if a gas embolism occurred during laparoscopy, with the risk of paradoxical embolism through the fenestration.

The feasibility of laparoscopy versus laparotomy was the key question of our multidisciplinary approach to managing this patient's condition. On the one hand, the benefits of laparoscopic surgery for this patient included a significant reduction in postoperative pain, length of hospital stay and likelihood of respiratory complications [8]. On the other hand, control of PVR and SVR during laparoscopy after pneumoperitoneum can be challenging, which would in turn make it difficult to maintain haemodynamic stability during the procedure.

Our peri‐operative strategy was designed to allow a quick return to spontaneous ventilation, to rapidly restore the beneficial effect of negative intrathoracic pressures during inspiration. We were fully aware that the pulmonary pressures would rise with IPPV and abdominal insufflation. The question was: how much is too much? No simple answer to that question can be found in the literature. It is known that long‐standing elevated mean pulmonary pressures are associated with decreased overall survival [9].

In this case, we decided to accept the risk of a short duration of high mean pulmonary pressures but monitored the balance of flows through the lungs and fenestration with S_p_O_2_ and blood‐gas analysis. We decided to maintain systemic perfusion pressure over 40 mmHg: calculated as mean arterial pressure minus mean pulmonary pressure. We closely monitored NIRS, urine output and serum lactate. Any drop in NIRS of more than 20%, urine output below 0.5 ml.kg.h^−1^ or elevation of lactate to more than 2 mmol.l^−1^ were considered signs of inadequate systemic perfusion or oxygen transport to end organs and used to trigger conversion to open surgery.

The postoperative period was complicated by respiratory distress despite non‐invasive ventilation. The predictable decrease of lung compliance induced by the Trendelenburg position and abdominal insufflation may have played an important role in this. Inhaled nitric oxide is one of the most potent pulmonary vasodilators as demonstrated by Roberts in 1993 [10]. In our case, NO_i_ initiation at arrival in the PICU worked well to improve PVR. In the context of failing Fontan, where the physiology is already pushed to the limits of viability, it may be advisable to consider initiating NO_i_ early in the procedure, possibly at induction of anaesthesia. We recommend that this drug not be limited to rescue therapy, as it might foster flow through the lungs by decreasing PVR, limiting venous systemic congestion and fluid overload.
